# Spherical MgSiO_3_–NH_2_ Adsorbents with Optimized Surface Chemistry for Humidity-Enhanced Direct Air CO_2_ Capture

**DOI:** 10.3390/ma19030588

**Published:** 2026-02-03

**Authors:** Sungho Park, Hyeok-Jung Kim

**Affiliations:** 1 Department of Biomedical Science, Daejin University, 1007, Hoguk-ro, Pocheon-si 11159, Gyeonggi-do, Republic of Korea; shopark@daejin.ac.kr; 2 Division of Architecture Engineering and Civil Engineering, Hoseo University, 20, Hoseo-ro 79 beon-gil, Baebang-eup, Asan-si 31499, Chungcheongnam-do, Republic of Korea

**Keywords:** direct air capture (DAC), spherical magnesium silicate, solid amine sorbents, humidity-enhanced CO_2_ capture, packed-bed pressure drop

## Abstract

Amine-functionalized solid adsorbents are widely recognized as promising candidates for direct air capture of CO_2_; however, their practical deployment remains constrained by humidity-dependent adsorption behavior and poor packed-bed operability arising from irregular particle morphology and fines generation. Rather than focusing solely on maximizing intrinsic adsorption capacity, this study addresses these process-level limitations through an integrated design strategy combining particle morphology control with surface chemistry optimization. Uniform spherical magnesium silicate particles with a mean diameter of approximately 15 μm were synthesized via a water-in-oil emulsion route to suppress fines formation and reduce hydrodynamic resistance. Controlled acid pretreatment was subsequently applied to adjust surface hydroxyl accessibility and enable efficient amine grafting without altering bulk composition. The optimized spherical magnesium silicate amine adsorbents exhibited pronounced humidity-enhanced carbon dioxide capture, achieving capacities of 1.7 to 1.8 millimoles/g at 50% relative humidity, representing an approximately fourfold increase compared with dry conditions. This enhancement is attributed to a humidity-induced mechanistic transition from carbamate formation under dry conditions to water-assisted bicarbonate formation under humid conditions. Complete regeneration was achieved at 100 °C, with stable adsorption desorption behavior maintained over ten consecutive cycles, demonstrating short-term reversibility. These findings highlight morphology controlled scalability. Future work should prioritize durability beyond 100 cycles, mechanical robustness, and techno-economic viability at scale.

## 1. Introduction

Amine-functionalized solid adsorbents have been widely investigated as promising materials for direct air capture (DAC) of CO_2_ because they offer high chemical selectivity toward CO_2_ and enable regeneration at relatively low temperatures compared to conventional carbonate- or oxide-based sorbents [1,2,3]. Recent studies on aminosilane-grafted silica and hierarchical mesoporous supports have demonstrated effective CO_2_ capture from both simulated and ambient air streams, highlighting the versatility of solid amine platforms for DAC applications [4,5,6]. A critical advantage of solid amine sorbents is their strong sensitivity to humidity. Under humid conditions, cooperative interactions between CO_2_ and water molecules promote bicarbonate formation, leading to enhanced adsorption capacity and faster uptake kinetics compared to dry environments [7,8,9]. This behavior has been consistently observed across different amine chemistries and support materials, underscoring the importance of moisture effects in realistic DAC operation [1,10].

Despite these favorable adsorption characteristics, the practical implementation of DAC remains challenging. Beyond intrinsic adsorption capacity, sorbent performance is strongly influenced by particle morphology, surface chemistry, and gas–solid hydrodynamics under near-atmospheric operating conditions [11]. In particular, process-oriented assessments have emphasized that laboratory-scale uptake values alone are insufficient to predict performance in fixed-bed or cyclic DAC systems [12,13].

Irregular particle shapes and the generation of fines are especially problematic in packed-bed reactors. Such morphological deficiencies can lead to excessive pressure drop, non-uniform flow distribution, and particle attrition, directly increasing blower energy demand and operational costs [11,14]. These effects become more pronounced during long-term cyclic operation, where gradual mechanical degradation deteriorates bed permeability and compromises process stability [15,16].

Accordingly, recent reviews and process studies increasingly emphasize that DAC sorbent design must extend beyond maximizing adsorption capacity to include process-relevant properties such as particle uniformity, mechanical robustness, and flowability [7,12,14]. Explicit consideration of these parameters is essential for bridging the gap between material-level performance and scalable DAC deployment.

Emulsion-assisted synthesis has emerged as an effective strategy for producing spherical inorganic particles with controlled size and morphology [17,18,19]. In water-in-oil (W/O) emulsion systems, dispersed aqueous droplets act as confined microreactors, within which nucleation and growth proceed under spatial restriction. This droplet confinement suppresses uncontrolled aggregation and secondary nucleation, enabling the formation of monodisperse spherical particles with reduced fines content [17,18]. For silicate-based materials, particle morphology can be tuned independently of bulk solution chemistry by adjusting emulsion stability, surfactant concentration, interfacial tension, stirring conditions, and precursor diffusion kinetics within the droplets [18,19]. Such decoupling of particle-scale morphology from intrinsic material composition is particularly advantageous for process-oriented DAC applications.

In magnesium silicate systems, emulsion-derived synthesis offers additional benefits by decoupling precipitation kinetics from bulk solution conditions. This localized reaction environment promotes homogeneous incorporation of Mg species into the silicate framework while suppressing the formation of irregular aggregates and nanoscale fines commonly observed in conventional precipitation or sol–gel routes [18]. Previous studies on emulsion-derived silicates and oxides have shown that droplet-confined precipitation can significantly improve particle uniformity, packing reproducibility, and flowability in packed-bed configurations [17,19]. However, systematic investigations of spherical magnesium silicate particles specifically optimized for amine functionalization and DAC-relevant packed-bed operation remain limited. In particular, the interplay between emulsion-derived morphology, surface hydroxyl chemistry, and subsequent amine grafting efficiency has not been fully elucidated.

Amine-based CO_2_ sorbents have been developed on a wide range of inorganic supports, including mesoporous silica, alumina, metal oxides, and hybrid materials, each presenting distinct trade-offs in adsorption performance and processability [1,2,11]. Mesoporous silica supports offer high surface area and tunable pore architectures but often suffer from irregular particle morphology and mechanical fragility, leading to fines generation and elevated pressure drop during packed-bed operation [2,11]. Metal oxides such as MgO and CaO exhibit strong intrinsic CO_2_ affinity; however, their practical application is frequently limited by poor amine dispersion, high regeneration energy penalties, or structural degradation under humid cycling conditions [16,20].

To place the present work in the context of recent DAC materials research, representative sorbent strategies reported in the literature are summarized in Table 1, with emphasis on their performance under humid conditions and practical packed-bed operability. As summarized in Table 1, while substantial progress has been made in enhancing intrinsic CO_2_ capture performance, unresolved challenges remain in amine utilization efficiency, flow resistance, and long-term operability under realistic DAC conditions.

In this context, the present study does not aim to maximize intrinsic adsorption capacity alone, but rather to address the often-overlooked limitations associated with packed-bed operability by integrating spherical particle morphology with chemically stable amine anchoring on magnesium silicate supports. Incorporation of Mg into the silicate framework increases accessible surface area and enhances amine anchoring stability through Lewis acid–base interactions, improving resistance to amine volatilization and leaching under humid conditions [20,25]. Simultaneously, spherical and monodisperse particle morphology improves bed packing uniformity, gas permeability, and mechanical durability, thereby reducing hydrodynamic resistance and susceptibility to attrition in fixed-bed reactors [13,14].

From a techno-economic perspective, the practical viability of solid amine sorbents for DAC is governed not only by adsorption capacity but also by material cost, process complexity, and energy penalties associated with gas transport and thermal regeneration [12,13]. Magnesium silicate supports benefit from widely available, low-cost precursors, while emulsion-assisted synthesis introduces additional processing steps related to surfactant handling and washing. These additional steps may be offset by the formation of uniform, fines-free particles that substantially reduce packed-bed pressure drop and associated blower energy consumption, a major contributor to DAC operating costs.

Motivated by these considerations, the present study combines emulsion-assisted synthesis of spherical MgSiO_3_ with controlled acid pretreatment to rationally tune surface hydroxyl density and optimize amine grafting efficiency. Rather than competing solely on maximum adsorption capacity, this work emphasizes a process-relevant materials design strategy that integrates particle morphology, surface chemistry, and packed-bed operability. By systematically linking emulsion-derived morphology, surface hydroxyl chemistry, and CO_2_ adsorption behavior under humid conditions, this study elucidates key structure–property relationships governing practical DAC performance and provides insights into the design of scalable solid amine adsorbents that balance capture efficiency, flow dynamics, and energy requirements.

## 2. Materials and Methods

### 2.1. Materials

All reagents were of analytical grade and used as received unless otherwise noted. Magnesium nitrate hexahydrate (Mg(NO_3_)_2_·6H_2_O, ≥99%) and sodium silicate solution (Na_2_SiO_3_, reagent grade) were purchased from Duksan Pure Chemicals (Ansan-si, Republic of Korea). Low-odor kerosene (≥99%, Daejung Chemicals, Siheung-si, Republic of Korea) was used as the continuous oil phase, and Span^®^ 80 (sorbitan monooleate, ≥99%, Sigma-Aldrich, St. Louis, MO, USA) served as the surfactant for emulsion formation. Ammonium bicarbonate (NH_4_HCO_3_, ≥99%, Sigma-Aldrich) was used as a buffering agent to facilitate controlled precipitation. Hydrochloric acid (HCl, 37 wt%, analytical grade, Daejung Chemicals), dimethyl sulfoxide (DMSO, ≥99.5%, Sigma-Aldrich), and 3-aminopropyltriethoxysilane (APTES, ≥98%, Sigma-Aldrich) were used for surface pretreatment and amine grafting. Ethanol (≥99.9%, Duksan Pure Chemicals) and deionized water were used for washing and purification. Ninhydrin (≥99%, Sigma-Aldrich) was used for quantitative determination of surface amine content. High-purity CO_2_ and N_2_ gases (99.999%) were supplied by Daesung Gas Co. (Pocheon-si, Republic of Korea). To ensure reproducibility, all key synthesis and functionalization procedures were performed through multiple independent repetitions. For each independently prepared sample, adsorption measurements, pressure-drop evaluations, and ninhydrin assays were conducted multiple times. Only minor variation was observed across repeated analyses, confirming the robustness of the reported trends. Representative values are therefore reported throughout this work.

### 2.2. Synthesis of Spherical MgSiO_3_ Particles

Spherical MgSiO_3_ particles were synthesized via a water-in-oil (W/O) emulsion method to achieve uniform morphology and narrow size distribution. The aqueous precursor solution was prepared by mixing sodium silicate and magnesium nitrate (0.25 M) under vigorous stirring. This aqueous phase was then slowly introduced into kerosene containing 3 wt% Span 80 homogenized at 100 rpm using a mechanical stirrer to form stable microdroplets. After homogenization, ammonium bicarbonate solution (0.1 M) was added dropwise to initiate magnesium silicate precipitation within the dispersed droplets. The reaction proceeded for 2 h at 60 °C under continuous stirring.

The resulting particles were separated by centrifugation and washed repeatedly with ethanol and deionized water to remove residual surfactant and ions. After overnight drying at 80 °C, the solids were calcined in air at 550 °C for 3 h to obtain porous spherical MgSiO_3_ particles with a mean diameter of 10–20 μm, as determined by laser diffraction.

For comparison, precipitation-derived MgSiO_3_ was prepared by directly mixing aqueous Mg(NO_3_)_2_ and sodium silicate solutions under alkaline conditions, followed by aging, filtration, drying, and calcination under identical conditions.

### 2.3. Acid Pretreatment for Surface Hydroxylation

The MgSiO_3_ spheres were subjected to acid pretreatment to increase surface silanol (Si–OH) density and enhance APTES grafting efficiency. Approximately 20 g of MgSiO_3_ powder was dispersed in 200 mL of HCl solution (0–2.0 M in 0.5 M increments) and stirred for 1 h at room temperature. The treated powders were then filtered, thoroughly washed with deionized water until the filtrate reached neutral pH, and dried at 100 °C.

Preliminary base treatments using NaOH solution (0–0.5 M) resulted in structural instability during post-treatment handling and washing, and were therefore not pursued further. Subsequent optimization focused exclusively on acid pretreatment to modulate surface hydroxyl accessibility.

### 2.4. Amine Functionalization via APTES Grafting

APTES was grafted onto hydroxylated MgSiO_3_ to introduce amine groups (–NH_2_) as CO_2_ chemisorption sites. In a typical procedure, 5 g of acid-treated MgSiO_3_ was dispersed in 100 mL of DMSO containing 5–10 wt% APTES. The suspension was heated to 90 °C and maintained at this temperature for 6 h under continuous stirring. After the reaction, the solids were collected by centrifugation, washed several times with ethanol to remove unreacted silane, and dried at 80 °C overnight. This study systematically compared the effects of acid pretreatment conditions and synthesis routes on achievable amine loading.

### 2.5. Characterization

Particle morphology and size distribution were examined using field-emission scanning electron microscopy (FE-SEM, JEOL JSM-7610F, JEOL, Tokyo, Japan) and laser diffraction (HELOS, Sympatec GmbH, Clausthal-Zellerfeld, Germany). Elemental composition analysis was performed using energy-dispersive X-ray spectroscopy (EDX, X-Max, Oxford Instruments, Abingdon, United Kingdom) coupled to the FE-SEM. Textural properties—including BET surface area, pore volume, and pore diameter—were determined from N_2_ adsorption–desorption isotherms at 77 K using a Micromeritics TriStar II analyzer(TriStar II, Micromeritics Instrument Corporation, Norcross, GA, USA). To ensure fair comparison between different synthesis routes, SEM and EDX analyses were conducted for both spherical and precipitation-derived MgSiO_3_ samples. Within the investigated acid pretreatment range, EDX analysis confirmed that the Mg/Si atomic ratio remained essentially unchanged, indicating that acid treatment did not induce measurable bulk compositional variation. CO_2_ adsorption measurements were conducted using a gravimetric DVS Carbon system (Surface Measurement Systems, London, United Kingdom), operated by MCC Korea Co., Ltd., Seoul, Republic of Korea, which enables precise monitoring of mass change under controlled humidity, gas flow, and cycling conditions. providing quantitative data on mass changes and humidity-dependent sorption kinetics.

Instrument calibration, buoyancy correction, and baseline subtraction procedures were performed in accordance with the manufacturer’s standard protocols, as documented in the DVS Carbon technical manual and commonly adopted in recent DAC studies.

### 2.6. Amine Loading Quantification

Surface amine content was quantified using a ninhydrin colorimetric assay [26]. Approximately 20 mg of MgSiO_3_–NH_2_ was dispersed in 10 mL of ethanol containing 0.5 wt% ninhydrin and heated to 80 °C for 15 min. After cooling, the supernatant was analyzed at 570 nm using a UV–Vis spectrophotometer (V-730, JASCO, Tokyo, Japan). Calibration with ethylamine standards was used to determine the amine concentration, which is reported as mmol–NH_2_ per gram of adsorbent. All ninhydrin assays were conducted through multiple independent repetitions for each sample to ensure reproducibility, and only minor variation was observed among repeated measurements. These measurements were used to systematically evaluate the influence of acid pretreatment concentration and synthesis route on achievable amine grafting efficiency.

### 2.7. CO_2_ Adsorption Performance Testing

CO_2_ capture performance was evaluated under both static and dynamic conditions. For static tests, approximately 100 mg of sample was loaded into a thermogravimetric analyzer and exposed to a CO_2_/N_2_ mixture (400 ppm CO_2_) at 25 °C and 1 atm. Continuous mass monitoring determined equilibrium uptake (mmol/g).

Detailed adsorption analysis was conducted using a gravimetric DVS Carbon system (Surface Measurement Systems), operated by MCC Korea Co., Ltd., to monitor CO_2_ sorption under controlled humidity and gas flow condition. Adsorbents were exposed to alternating dry and humidified CO_2_ streams (0% and 50% relative humidity) to investigate moisture effects on CO_2_ capture behavior. Breakthrough experiments were performed to assess adsorption kinetics and uptake capacity under continuous flow conditions. Calibration, buoyancy correction, and baseline subtraction procedures were conducted following the manufacturer’s standard protocols, as documented in the DVS Carbon technical manual and commonly adopted in recent DAC studies [27]. Regeneration behavior was evaluated by switching the gas steam to high-purity N_2_ at 100 °C, followed by repeated adsorption–desorption cycles to assess short-term cyclic stability. The DVS system simultaneously monitored mass changes and gas composition, enabling quantitative evaluation of humidity-dependent uptake, regeneration efficiency, and cyclic durability [6,15].

### 2.8. Packed-Bed Pressure Drop Evaluation

Pressure drop (ΔP) and flow behavior were evaluated using a fixed-bed apparatus that simulated DAC-relevant gas–solid contact conditions. A cylindrical column with an inner diameter of 20 mm and packed-bed height of 150 mm was used for all measurements. Adsorbents were packed using gentle vibration to achieve consistent packing density and minimize bed heterogeneity.

Two materials were compared: (i) spherical MgSiO_3_ synthesized in this study with a mean diameter of approximately 15 μm, and (ii) precipitation-derived MgSiO_3_ with a similar mean diameter but a broad size distribution containing a significant fraction of fines. CO_2_/N_2_ carrier gas was introduced at controlled flow rates ranging from 0.025 to 0.150 L/min, corresponding to superficial velocities of 0.01–0.06 m/s. A differential pressure transducer was used to measured ΔP across the packed bed, and steady-state values were recorded after system stabilization. All measurements were conducted at ambient temperature [9,27].

All pressure-drop experiments were performed under tightly packed-bed conditions, and the investigated gas velocities were selected to reflect practical DAC operation rather than to approach incipient fluidization. Accordingly, the reported pressure-drop data are intended to enable relative comparison of morphology-dependent flow resistance between spherical and precipitation-derived MgSiO_3_, rather than to provide absolute fluid-dynamic parameters such as minimum fluidization velocity or Ergun-type permeability coefficients. Each pressure-drop measurement was repeated multiple times for independently prepared beds to confirm reproducibility, and representative values are reported based on consistent trends observed across repeated experiments.

## 3. Results

### 3.1. Synthesis and Characterization of Spherical MgSiO_3_

**Figure 1:**
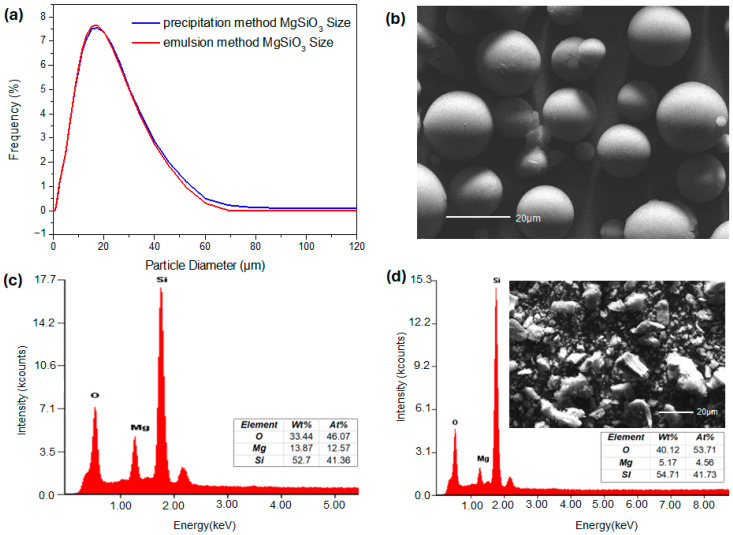
(**a**) Particle size distribution, (**b**) SEM morphology, and (**c**) EDX composition of spherical MgSiO_3_ synthesized via emulsion-assisted precipitation; (**d**) SEM morphology and EDX composition of precipitation-derived MgSiO_3_.

SEM imaging (Figure 1) confirmed that the emulsion-assisted synthesis produced MgSiO_3_ particles with highly uniform spherical morphology and smooth, defect-free surfaces. This monodisperse particle formation reflects the stability of emulsion droplets during nucleation and growth, distinguishing the material from precipitation- or sol–gel-derived silicates that typically contain irregular aggregates and nanosized fines. The resulting fines-free spherical architecture offers critical advantages for packed-bed applications, where reproducible packing and stable gas flow are essential.

Particle-size analysis revealed a unimodal distribution, with most particles ranging from 10 to 25 μm and a mean diameter of approximately 15 μm. This narrow distribution creates a high and consistent void fraction within packed beds, minimizing flow resistance and enabling the low pressure drop observed in subsequent evaluations. The absence of nanoscale debris prevents void clogging and ensures uniform gas transport—a key requirement for DAC systems that depend on steady airflow through the sorbent bed.

EDX analysis confirmed the expected stoichiometric composition (Si: 41.36 at%, O: 46.07 at%, Mg: 12.57 at%), demonstrating chemical uniformity and structural integrity across all spheres. This compositional stability indicates that the material provides a reliable platform for amine grafting without undergoing undesired chemical transformations during functionalization.

For comparison, precipitation-derived MgSiO_3_ was also characterized by SEM and EDX to evaluate morphological and compositional differences relative to the emulsion-synthesized material. As shown in Figure 1, the precipitation route produced irregularly shaped particles with comparable mean particle size but markedly lower morphological uniformity, accompanied by the presence of fine particles, in contrast to the uniform spherical morphology obtained via emulsion synthesis. EDX analysis further revealed a lower Mg/Si atomic ratio for the precipitation-derived MgSiO_3_ compared to the spherical material, indicating compositional differences in addition to morphological variation. These morphological and compositional differences are expected to influence packed-bed hydrodynamics and process-level performance, as discussed in Section 3.5.

The combination of spherical morphology, controlled size distribution, and homogeneous composition establishes emulsion-derived MgSiO_3_ as both mechanically and chemically robust. These properties enhance flowability, reduce hydrodynamic resistance, and ensure reproducible surface functionalization—collectively positioning the material as an effective support for amine-based CO_2_ capture.

### 3.2. Effect of Acid Pretreatment on Surface Hydroxylation

**Figure 2:**
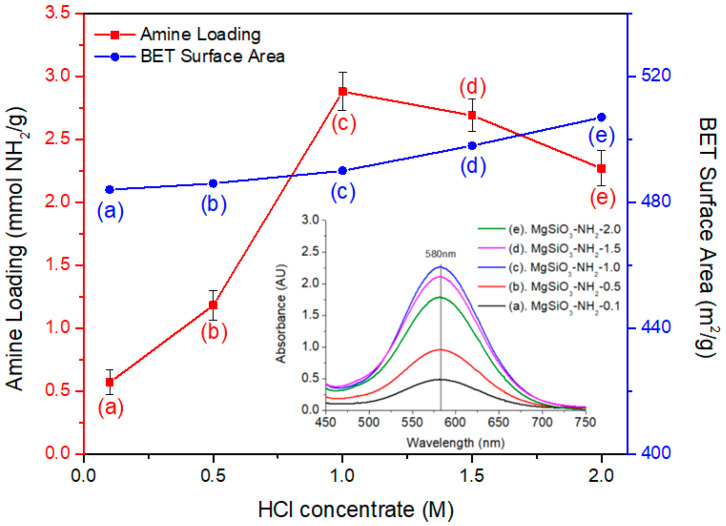
BET surface area and amine loading of spherical MgSiO_3_ as a function of HCl pretreatment concentration. (a) MgSiO_3_-NH_2_-0.1; (b) MgSiO_3_-NH_2_-0.5; (c) MgSiO_3_-NH_2_-1.0; Moderate acid treatment (1.0 M) yields the highest amine loading despite only a slight increase in surface area. (d) MgSiO_3_-NH_2_-1.5 (e) MgSiO_3_-NH_2_-2.0.

Figure 2 illustrates how HCl concentration during pretreatment influences the structural and chemical properties of spherical MgSiO_3_. BET surface area increased moderately from 445 m^2^/g at 0.1 M HCl to approximately 515 m^2^/g at 2.0 M, indicating that controlled acid treatment progressively modifies the surface structure without compromising particle integrity. In contrast, amine loading exhibited a pronounced volcano-type dependence on acid concentration, reaching a maximum of 2.889 mmol–NH_2_/g at 1.0 M HCl (determined by ninhydrin colorimetry). As the HCl concentration increased, the BET surface area of MgSiO_3_ gradually increased, whereas the amine loading exhibited a clear maximum at 1.0 M and decreased at higher acid concentrations. EDX analysis confirmed that the Mg/Si atomic ratio remained essentially unchanged across the investigated HCl concentration range.

This optimal amine loading arises from balanced generation of reactive surface silanol (≡Si–OH) groups during moderate acid treatment. At low acid concentrations, hydrolysis of the MgSiO_3_ framework is insufficient to generate the silanol density required for efficient APTES condensation. As the HCl concentration increases to 1.0 M, controlled surface activation exposes additional silanol groups while preserving the structural integrity of the framework, thereby enabling effective aminosilane grafting. At higher concentrations (up to 1.5 M HCl), no measurable Mg^2+^ leaching was detected, and the Mg/Si atomic ratio remained unchanged within experimental error, indicating that extensive proton-assisted cleavage of Si–O–Mg linkages does not occur under the present pretreatment conditions. Accordingly, the increase in BET surface area observed after acid treatment is not attributed to Mg dissolution or the formation of a silica-rich gel layer. Instead, the surface area increase is interpreted as arising from subtle surface restructuring and enhanced structural flexibility induced by protonation of metal–oxygen bonds (e.g., Si–O–Mg and Si–O–Si). Proton incorporation can weaken local bonding interactions and modify near-surface connectivity, allowing partial relaxation or rearrangement of the surface structure and increasing apparent surface accessibility without altering the bulk composition of MgSiO_3_.

However, this structural modification does not necessarily translate into a higher density of reactive silanol groups available for APTES grafting. Although the samples exhibit mesoporous characteristics with pore volumes in the range of approximately 0.23~0.55 cm^3^/g and dominant pore sizes spanning 5~40 nm, no systematic correlation was observed between these pore parameters and amine loading across the investigated HCl concentration range. As a result, amine loading does not continue to increase at higher HCl concentrations despite the monotonic increase in BET surface area. This interpretation is therefore proposed as a mechanistic hypothesis, consistent with the observed compositional stability and the experimentally measured trends in surface area, pore characteristics, and amine loading, rather than as a definitive structural assignment.

These results demonstrate that silanol availability and reactivity—rather than total surface area—primarily govern amine grafting efficiency for spherical MgSiO_3_. Moderate acid pretreatment at 1.0 M HCl therefore provides an optimal balance between framework activation and structural preservation, enabling maximum amine loading for CO_2_ capture. 

### 3.3. CO_2_ Adsorption Capacity: Spherical vs. Precipitated MgSiO_3_

**Figure 3:**
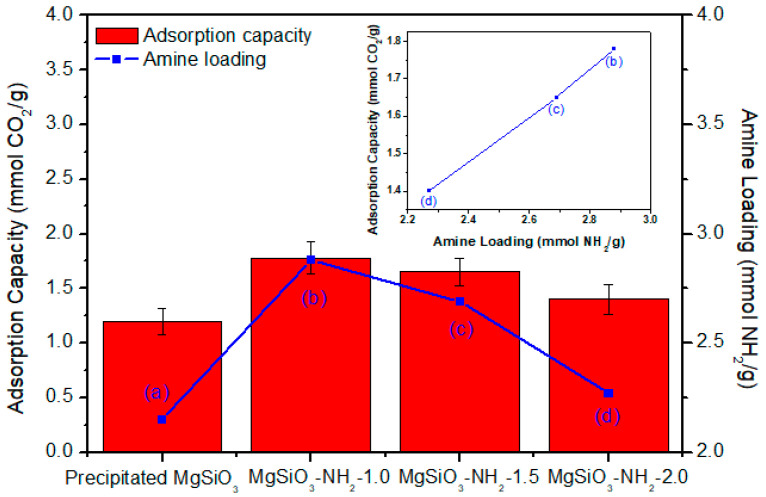
CO_2_ adsorption capacities of spherical MgSiO_3_–NH_2_ samples prepared under different HCl pretreatment concentrations, compared with precipitated MgSiO_3_. MgSiO_3_–NH_2_-1.0 exhibits the highest uptake, reflecting its optimal amine loading and accessible surface area. The inset illustrates the relationship between amine loading and adsorption capacity.

Figure 3 compares the CO_2_ adsorption capacities of amine-functionalized spherical MgSiO_3_ samples prepared under varying HCl pretreatment conditions with a precipitation-derived reference material. Although the precipitated MgSiO_3_ exhibited a comparable mean particle size of approximately 15 μm, its compositional and textural properties differed substantially from those of the emulsion-derived spheres. Notably, precipitation-derived MgSiO_3_ contained only about half of the Mg content of the spherical material, resulting in a significantly lower BET surface area (~250 m^2^/g). This reduced surface area limited the number of accessible silanol groups available for APTES grafting, leading to lower amine loading and the weakest CO_2_ uptake among all samples (≈1.2 mmol/g).

In contrast, spherical MgSiO_3_–NH_2_ materials demonstrated consistently higher CO_2_ adsorption capacities across all pretreatment conditions. The sample pretreated with 1.0 M HCl achieved the highest amine loading (2.889 mmol–NH_2_/g) and the highest CO_2_ uptake (≈1.8 mmol/g). Samples treated with higher acid conditions (1.5 M and 2.0 M HCl) exhibited slightly reduced CO_2_ capacities (≈1.7 and 1.6 mmol/g, respectively), reflecting their correspondingly lower amine loadings. The untreated sample (0 M HCl) showed the lowest amine loading and CO_2_ uptake among the spherical materials, confirming that acid activation is essential for generating surface functionality required for effective APTES grafting.

Figure 3 further reveals a near-linear correlation between amine loading and CO_2_ adsorption capacity for both spherical and precipitation-derived MgSiO_3_. This direct proportionality confirms that grafted amine density is the dominant factor governing CO_2_ uptake, while differences between emulsion and precipitation synthesis routes primarily manifest through their influence on accessible surface sites and resultant amine loading.

To further clarify the relationship between surface functionalization and adsorption performance, an additional representation in Figure 3 presents CO_2_ adsorption capacity as a function of amine loading for both spherical and precipitation-derived MgSiO_3_ samples. A near-linear correlation is observed across all samples, reinforcing that accessible amine density—rather than particle morphology or surface area alone—is the primary determinant of CO_2_ uptake under humid DAC-relevant conditions.

### 3.4. CO_2_ Adsorption Performance

The CO_2_ adsorption behavior and cyclic durability of MgSiO_3_–NH_2_ samples were evaluated using a DVS Carbon system under controlled humidity and flow conditions. Preliminary measurements conducted under dry conditions (0% RH) showed relatively low CO_2_ uptake, whereas all samples exhibited substantial enhancement upon introduction of humidity, consistent with moisture-assisted amine chemisorption. Samples with higher amine loadings (HCl-treated series) displayed correspondingly higher CO_2_ uptake and faster absorption kinetics. The following sections describe absorption kinetic under dry conditions, humidity-enhanced CO_2_ capture behavior, and cyclic stability.

#### 3.4.1. Dry-Condition Adsorption Kinetics

**Figure 4:**
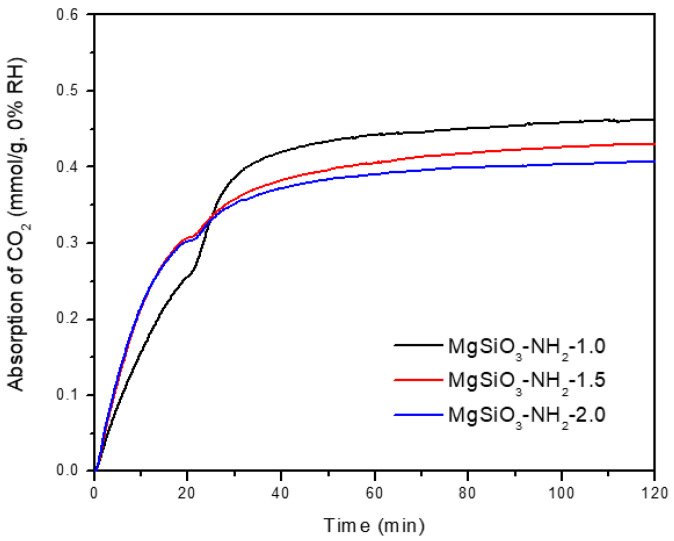
CO_2_ adsorption profiles of MgSiO_3_–NH_2_ samples prepared via HCl pretreatment and evaluated under dry conditions (0% RH).

The adsorption kinetics of MgSiO_3_–NH_2_ samples pretreated with 1.0 M, 1.5 M, and 2.0 M HCl under dry conditions (0% RH) are presented in Figure 4. All three adsorbents exhibited rapid initial uptake during the first 20–30 min, followed by a gradual approach to equilibrium over approximately 120 min. This two-stage absorption profile reflects fast surface chemisorption on amine groups followed by slower diffusion-controlled uptake within the mesoporous pore network.

Despite differences in amine loadings—2.889 mmol/g (1.0 M), 2.266 mmol/g (1.5 M), and 2.228 mmol/g (2.0 M)—the equilibrium CO_2_ uptake remained relatively low under dry conditions. This behavior is attributed to the dominant carbamate formation mechanism, which requires two amine groups per CO_2_ molecule (1:2 stoichiometry) and proceeds with relatively high activation energy [10]. As a result, only spatially accessible amine pairs participate in chemisorption, leaving significant fraction of grafted amines unutilized and yielding equilibrium capacities of approximately 0.40–0.50 mmol/g.

The 1.0 M HCl-treated sample achieved the highest equilibrium uptake (≈0.45–0.50 mmol/g), consistent with its higher amine loading. Samples treated with 1.5 M and 2.0 M HCl showed slightly lower capacities (≈0.40–0.45 mmol/g). Notably, the 1.0 M sample exhibited a more gradual initial uptake slope despite reaching the highest final capacity, indicating that early-stage adsorption kinetics and equilibrium uptake are governed by different factors. Early uptake reflects surface reactivity and accessibility, whereas final capacity depends primarily on the total density of amine sites available for carbamate formation.

#### 3.4.2. Humidity-Enhanced CO_2_ Capture

**Figure 5:**
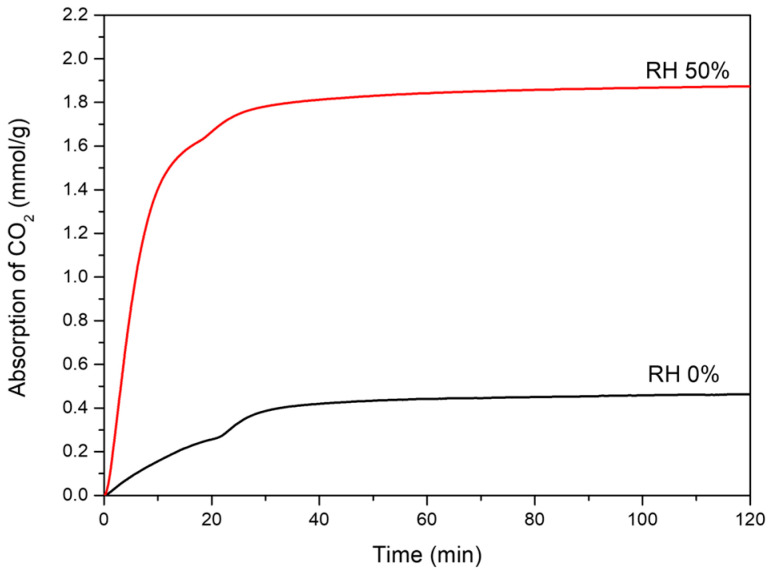
Humidity-dependent CO_2_ adsorption behavior of MgSiO_3_–NH_2_-1.0, which contained 2.889 mmol NH_2_/g (ninhydrin colorimetry), measured at 25 °C under 0% and 50% relative humidity.

To evaluate the effect of moisture on adsorption performance, MgSiO_3_–NH_2_ pretreated with 1.0 M HCl was tested under both dry (0% RH) and humid (50% RH) conditions, as shown in Figure 5. Under dry conditions, equilibrium CO_2_ uptake was limited to approximately 0.35–0.40 mmol/g, with the adsorption reaching a plateau after 60 min. In contrast, at 50% RH, CO_2_ uptake increased markedly to 1.7–1.8 mmol/g, representing an approximately fourfold enhancement. In addition, adsorption under humid conditions reached equilibrium within 20–30 min, indicating significantly accelerated kinetics.

This enhancement originates from a moisture-induced shift in the dominant adsorption mechanism. Under dry conditions, CO_2_ capture proceeds primarily via carbamate formation with 1:2 stoichiometry and relatively high activation energy. In the present of water, however, bicarbonate formation becomes increasingly favorable, as CO_2_ reacts with adsorbed water to form HCO_3_^−^ species stabilized by protonated amines. Previous studies have reported that, under humid conditions, the bicarbonate formation pathway proceeds with a lower activation barrier than carbamate formation, enabling faster adsorption kinetics; although carbamate species may still form concurrently, the presence of water facilitates broader participation of accessible amine sites by lowering kinetic barriers and enhancing CO_2_ mobility within the adsorbent, leading to higher effective amine utilization [28,29,30].

The extended exposure at 50% RH likely allowed nearly all accessible amine sites to participate in CO_2_ capture. The resulting uptake of 1.7–1.8 mmol/g reflects adsorption behavior under conditions where bicarbonate formation dominates, while carbamate formation may occur as a secondary pathway [10,12]. These results demonstrate that moisture plays a critical role in activating efficient CO_2_ capture pathways and enabling high amine utilization under DAC-relevant conditions [31,32].

#### 3.4.3. Cyclic Stability and Regeneration

**Figure 6:**
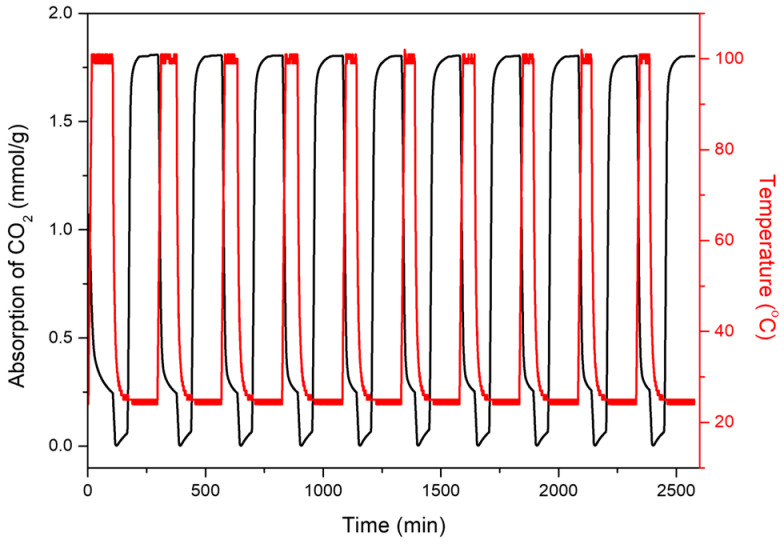
Ten-cycle CO_2_ adsorption–desorption stability of MgSiO_3_–NH_2_-1.0 measured using the DVS Carbon system.

The long-term stability of MgSiO_3_–NH_2_ (1.0 M) was evaluated through repeated adsorption–desorption cycling using the DVS Carbon system (Figure 6). Over ten consecutive cycles, the adsorbent exhibited highly reproducible adsorption profiles, with CO_2_ uptake rapidly increasing to approximately 1.7–1.8 mmol/g during each adsorption step at ambient temperature. During regeneration at 100 °C under N_2_, the initial mass decrease corresponds to the release of chemisorbed CO_2_ species, while the subsequent rapid drop reflects chamber purging and baseline correction. Both adsorption plateaus and desorption baselines overlapped closely across all cycles, with no measurable loss in capacity or distortion of adsorption profiles. The stable cyclic performance indicates that APTES-derived amine groups are covalently anchored through robust Si–O–Si linkages, allowing the adsorbent to withstand repeated CO_2_/H_2_O exposure without degradation or leaching. Overall, the DVS cycling results confirm that MgSiO_3_–NH_2_ (1.0 M) combines high CO_2_ capacity, fast adsorption–desorption kinetics, and excellent short-term durability, supporting its suitability for continuous DAC operation under humid conditions [10,14].

### 3.5. Pressure Drop and Flow Dynamics

Beyond surface chemistry, macroscopic particle morphology and size distribution play a critical role in determining process-level performance in packed-bed DAC systems. Although precipitation-derived MgSiO_3_ exhibited a comparable mean particle size of approximately 15 μm, its broad size distribution and substantial fines content resulted in significantly higher flow resistance. Fine particles readily occupied interparticle voids, constricting gas pathways and causing steep increased in pressure drop (ΔP) even at relatively low gas flow rates.

**Figure 7:**
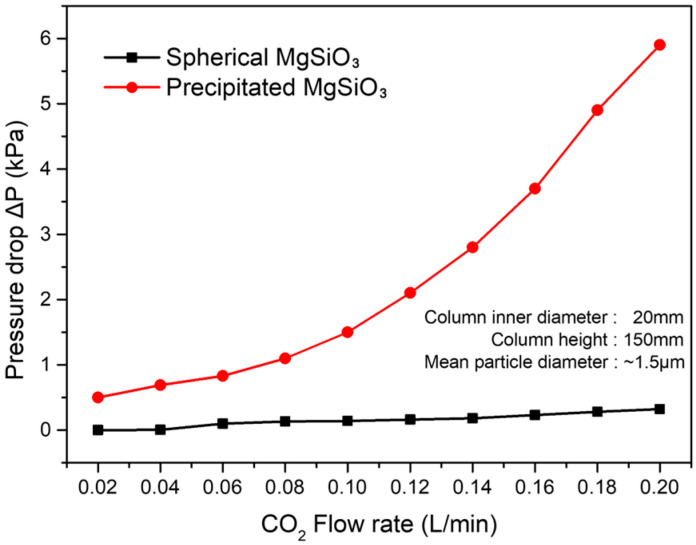
Comparison of pressure drop (ΔP) between spherical MgSiO_3_–NH_2_-1.0 (1.5 µm) and precipitated MgSiO_3_ containing fines, measured using a packed-bed column (inner diameter: 20 mm, bed height: 150 mm, superficial velocity: 0.01–0.06 m/s).

In contrast, uniform spherical MgSiO_3_ particles formed stable, highly permeable packed beds with well-defined void spaces [8,15]. This morphology enabled smooth gas transport, resulting in near-zero ΔP up to 0.10 L/min and only minor increases at higher flow rates, as shown in Figure 7. The markedly reduced pressure drop observed for the spherical MgSiO_3_ bed is attributed to the absence of fines and the narrow particle size distribution, which together minimize void blockage and promote uniform gas flow through the packed bed.

These results demonstrate that particle morphology and size uniformity are process-critical parameters that influence hydrodynamic resistance independently of adsorption capacity. The reduced pressure drop directly translates to lower blower-energy demand and improved operational efficiency for continuous DAC operation. Collectively, the combination of high CO_2_ uptake, rapid adsorption–desorption kinetics, excellent cyclic stability, and minimal hydrodynamic resistance establish amine-functionalized spherical MgSiO_3_ as a promising candidate for scalable, low-energy direct air capture applications [9,12,13,33].

### 3.6. Limitations and Future Perspectives for Practical DAC Deployment

The present study provides a proof-of-concept demonstration of morphology-controlled spherical MgSiO_3_–NH_2_ adsorbents for direct air capture applications. While the adsorption performance and short-term cyclic stability of the material were systematically evaluated, several limitations must be addressed to assess its practical applicability at larger scales.

Although cyclic stability was examined over ten consecutive adsorption–desorption cycles to confirm short-term reversibility and regeneration behavior, demonstration of long-term durability over hundreds or thousands of cycles will be required to establish commercial viability.

In addition, the adsorption experiments in this study were conducted under fixed temperature and humidity conditions to enable controlled comparison and mechanistic interpretation. However, real atmospheric environments are inherently dynamic, with continuous fluctuations in humidity and temperature across diurnal and seasonal cycles; future studies should therefore evaluate adsorption behavior under variable operating conditions that more closely simulate field deployment scenarios.

From a process-development perspective, while the emulsion-assisted synthesis route provides excellent control over particle size uniformity and spherical morphology, scale-up to industrially relevant volumes may introduce challenges related to processing cost, surfactant recovery efficiency, and wastewater treatment requirements. Transitioning toward continuous or semi-continuous emulsion processes represents a potential strategy to mitigate these challenges while preserving morphological control, and such approaches will be explored in future work alongside techno-economic assessment.

Finally, although the ~15 μm particle size achieved in this study is advantageous for reducing pressure drop and improving packed-bed operability, the mechanical robustness and attrition resistance of the porous spherical particles under prolonged gas flow were not systematically evaluated. Dedicated mechanical testing, including compression resistance and accelerated attrition protocols under DAC-relevant flow conditions, will be necessary to ensure long-term structural integrity and operability.

Addressing these material, process, and operational considerations will be essential for advancing spherical MgSiO_3_–NH_2_ adsorbents from laboratory-scale demonstration toward practical and scalable direct air capture deployment.

## 4. Conclusions

This study demonstrates that the integrated control of particle morphology and surface chemistry represents a critical design dimension for practical amine-based direct air capture (DAC) adsorbents. Moving beyond the sole pursuit of maximum intrinsic adsorption capacity, this work highlights how morphology-driven factors—specifically fines suppression, bed packing uniformity, and gas transport behavior—govern adsorbent performance under DAC-relevant operating conditions.

By employing a water-in-oil emulsion synthesis route, uniform spherical MgSiO_3_ particles with an optimized mean size of approximately 15 μm were successfully synthesized. This particle size was selected to balance surface accessibility with practical handleability in packed-bed configurations. The spherical morphology effectively mitigated fines formation and resulted in a markedly reduced pressure drop under application-relevant flow conditions. In parallel, controlled acid pretreatment enabled tuning of surface hydroxyl accessibility, facilitating efficient amine grafting without inducing structural instability. As a result, spherical MgSiO_3_–NH_2_ adsorbents exhibited pronounced humidity-enhanced CO_2_ capture, achieving capacities of 1.7–1.8 mmol/g at 50% RH, complete regeneration at a moderate temperature of 100 °C, and stable short-term cyclic performance. Beyond the specific material system investigated, these results underscore a broader design principle: effective DAC sorbents must be engineered at the particle level to balance accessible surface chemistry with favorable hydrodynamics and process operability, rather than optimizing adsorption capacity alone. Morphology-controlled supports, such as spherical MgSiO_3_, therefore represent a versatile and generalizable platform for the development of solid amine sorbents tailored for fixed-bed or modular DAC contactors.

While the present results demonstrate the potential of spherical MgSiO_3_–NH_2_ as a practical solid amine platform for DAC, several challenges remain prior to commercial deployment. In particular, long-term cyclic stability over hundreds to thousands of adsorption–desorption cycles, performance under dynamically fluctuating ambient conditions (including temperature, humidity, and trace atmospheric contaminants), mechanical durability under sustained industrial gas flow, and scalable, cost-effective synthesis strategies require systematic investigation. Addressing these material-, process-, and operation-level considerations will be essential for translating morphology-engineered solid amine adsorbents from laboratory-scale proof-of-concept demonstrations to energy-efficient, large-scale direct air capture systems.

## Figures and Tables

**Table 1:** Comparative overview of representative DAC sorbents strategies.

Strategy	Sorbent Type (Production Cost)	Regeneration Method (Operating Cost)	Key Advantages	Key Challenges	Key Review Citations
Amine-functionalized silica	Solid amine sorbents (PEI, TEPA on silica, alumina, or cellulose) (Low)	Temperature-swing (80–120 °C) (High)	High selectivity for CO_2_; tunable amine loading; moderate energy demand	Oxidative degradation; moisture sensitivity; amine leaching	[21]
Humidity-swing sorbents	Anion-exchange resins (quaternary ammonium functionalized) (Moderate)	Moisture-swing (30–80% RH) (Moderate)	Low-temperature regeneration (<100 °C); water-driven desorption	Requires humid climate; slow kinetics; limited capacity	[21]
Metal oxide nano-sorbents	Nanostructured CaO, MgO, K_2_CO_3_-promoted oxides (Moderte)	Temperature-swing (700–900 °C for CaO) or electrochemical (Very High)	High theoretical capacity (CaO: 17.8 mmol g^−1^); abundant materials	Very high regeneration temperature; sintering; slow carbonation	[22]
Structured/spherical sorbents	Amine-impregnated monoliths, beads, or 3D-printed structures (Moderate)	Temperature-swing or vacuum-swing (Low)	Reduced pressure drop; enhanced mass transfer; scalable contacting	Complex fabrication; mechanical stability; cost	[22]
MOFs/COFs	Amine-functionalized MOFs (e.g., mmen-Mg_2_(dobpdc)); imine-linked COFs (High)	Temperature-swing (60–140 °C) or vacuum-swing (High)	Ultra-high surface area; tunable pore chemistry; step-shaped isotherms	High synthesis cost; moisture instability; scale-up challenges	[23]
Alkali carbonate systems	K_2_CO_3_/Al_2_O_3_, Na_2_CO_3_/CaO composites (Low)	Temperature-swing (200–300 °C) or moisture-assisted (High)	Stable cycling; low-cost materials; integrated capture–conversion	Moderate regeneration temperature; slow kinetics; water management	[21]
Process engineering & system optimization	Integration of sorbents with renewable energy; AI/ML-driven optimization (Low)	Hybrid regeneration (solar, waste heat, electrochemical) (High)	Improved energy efficiency; cost reduction (>30% via renewables); accelerated material discovery	System complexity; infrastructure requirements; integration challenges	[22,24]
Spherical MgSiO_3_	Amine-grafted spherical magnesium silicate (~15 μm) (Low)	Temperature-swing (≈100 °C) (Low)	low flow resistance; fines-free packed-bed operability	Long-term durability (>1000 cycles)	This study

## Data Availability

The original contributions presented in this study are included in the article. Further inquiries can be directed to the corresponding author.
